# The complete chloroplast genome and phylogenetic analysis of *Nephelium lappaceum* (rambutan)

**DOI:** 10.1080/23802359.2021.1872449

**Published:** 2021-02-09

**Authors:** Jin Liu, Cheng Zheng, Zi-yan Liu, Ying-feng Niu

**Affiliations:** Yunnan Institute of Tropical Crops, Xishuangbanna, China

**Keywords:** *Nephelium lappaceum*, chloroplast genome sequence, phylogenetic analysis

## Abstract

*Nephelium lappaceum* is a popular tropical fruit belonging to the *Sapindaceae* family. The plant originated in Malaysia and Indonesia and is commonly called rambutan. Because of its refreshing flavor and exotic appearance, rambutan is widely accepted in the World. Due to its significant medicinal properties, the fruit has also been employed in traditional medicine for centuries. The chloroplast genome of rambutan was sequenced, assembled, and annotated in the present study. The chloroplast genome length was 161,356 bp and contained 132 genes, including 87 protein-coding genes, 37 transfer RNA (tRNA) genes, and 8 ribosomal RNA (rRNA) genes. It possessed the typical quadripartite circle structure with a large single-copy region (86,009 bp), a small single-copy region (18,153 bp), and two inverted repeat regions (28,597 bp). A total of 35 SSR markers were found in the chloroplast genome of Nephelium lappaceum, of which 33 were monomer, 1 was dimer and 1 was tetramer. Phylogenetic analysis based on the complete chloroplast genome sequences of 21 plant species showed that rambutan was closely related to *Pometia tomentosa*. These results provide a foundation for further phylogenetic and evolutionary studies of the *Sapindaceae* family.

*Nephelium lappaceum* Linn. 1767, commonly called rambutan, is a popular tropical fruit of the *Sapindaceae* family that originated in Malaysia (Wall 2006; Odey 2012). In terms of phylogenetic relationship, rambutan is closely related to lychee, pulasan, and longan (Greenand Popenoe 1975). Although rambutan is a fruit of Asian origin, it is widely appreciated in other parts of the World due to its refreshing flavor and exotic appearance, where it is consumed fresh, canned, or processed (Almeyada et al. 1979). Rambutan also has medicinal properties (Soeng et al. 2015; Mahmood et al. 2018); the rind extract exhibits high anti-oxidant (Palanisamy et al. 2008; Nurhuda et al. 2013), antibacterial (Thitilertdecha et al. 2010; Yuvakkumar et al. 2014), anti-Herpes Simplex virus type 1 (Nawawi et al. 1999) and anti-hyperglycemic (Palanisamy et al. 2011) activities. In Southeast Asia, the dried fruit rind has been employed in traditional medicine for centuries (Phang et al. 2019).

Despite its significance, genomics research on rambutan is very little. The chloroplast genome contains a large amount of genetic information related to various biological processes such as photosynthesis, which is crucial in studying plant evolution, genetic relationships, and accurate evaluation of germplasm resources (Niu et al. 2020). This study is the first report on the sequencing, assembly, and annotation of the rambutan chloroplast genome.

Young leaves of rambutan were collected from the Xishuangbanna Tropical Flowers and Plants Garden (100.786425 E, 22.011135 N), Yunnan province of China, and the specimen was deposited at the herbarium of the Yunnan Institute of Tropical Crops (http://www.yitc.com.cn/, fzzx@yitc.com.cn) under the voucher number of YITC-2020-FZ-S-006. The genomic DNA was extracted by a DNeasy Plant Mini Kit (Qiagen. Hilden, Germany), and the DNA quality was evaluated by a Nano-Drop 2000 spectrometer (Thermo Fisher Scientific, Waltham, MA, USA). DNA library for sequencing was constructed with insert sizes of 350 bp, and paired-end (PE) sequencing was conducted on the Illumina HiSeq 2500 platform (Illumina, San Diego, CA, USA). Subsequently, approximately 7.5 Gb of raw data were obtained and then assembled by GetOrganelle (Jin et al. 2020). The chloroplast genome was annotated by GeSeq (Tillich et al. 2017), examined manually by Geneious 11.1.5 (Kearse et al. 2012), and submitted to the GenBank under accession number MT884002.

The length of the rambutan chloroplast genome was 161,356 bp with a 37.78% GC content, and the genome structure was relatively comparable to that of other plant species (Liu et al. 2018). It possessed the typical quadripartite circle structure with two identical copies of inverted repeat regions (IRs, 28,597 bp) separated by a large single-copy region (LSC, 86,009 bp) and a small single-copy region (SSC, 18,153 bp). The GC contents of the LSC, IRs, and SSC regions were 36.03%, 42.28%, and 31.86%, respectively. The circular genome contained 132 genes, including 87 protein-coding genes, 37 tRNA genes, and 8 rRNA genes. The protein-coding genes are involved in photosystem I, photosystem II, the cytochrome b/f complex, ATP synthase, NADH dehydrogenase, RNA polymerase, and other biological functions. A total of 11 protein-coding genes, including *rps16*, *atpF*, *rpoC1*, *petB*, *petD*, *rpl16*, *rpl2*, *ndhB*, and *ndhA*, contained one intron, while four protein-coding genes, including *rps12*, *ycf3*, and *clpP*, contained two introns. The five smallest protein-coding genes were *petN*, *petL*, *psbM*, *psbT*, and *psbI*, and their sizes were 90 bp, 96 bp, 105 bp, 108 bp, and 111 bp. Meanwhile, the five largest protein-coding genes were *ycf2*, *ycf1*, *rpoC2*, *rpoB*, and *psaA*, and their sizes were 6855 bp, 5712 bp, 4182 bp, 3213 bp, and 2253 bp, respectively.

The MISA software (https://webblast.ipk-gatersleben.de/misa/) was used to analyze the microsatellite SSR, during the analysis, the minimum numbers of repeats were set to 10, 6, 5, 5, 5, and 5 for monomer, dimer, trimer, tetramer, pentamer, and hexamer, respectively. A total of 35 SSR markers were found in the chloroplast genome of Nephelium lappaceum, of which 33 were monomer, 1 was dimer and 1 was tetramer.

A maximum likelihood phylogenetic tree based on the complete chloroplast genome sequences of 22 plant species was constructed to ascertain the placement of rambutan within the Sapindaceae family. Twenty species belong to the family *Sapindaceae,* while the two species *Aglaia odorata* and *Cipadessa cinerascens* of the *Meliaceae* family was used as an outgroup. The 22 complete chloroplast genome sequences were aligned by MAFFT (Katoh and Standley 2013), and maximum likelihood analysis was performed by RAxMLbased on the GTRGAMMA substitution model (Stamatakis 2014) with 1000 bootstrap replicates. The result indicated that compared to other species of the *Sapindaceae* family, rambutan is more closely related to *Pometia tomentosa* (Figure 1). This study can provide a reference for developing markers and further research on the phylogeny and evolution of the *Sapindaceae* family.

**Figure 1.**
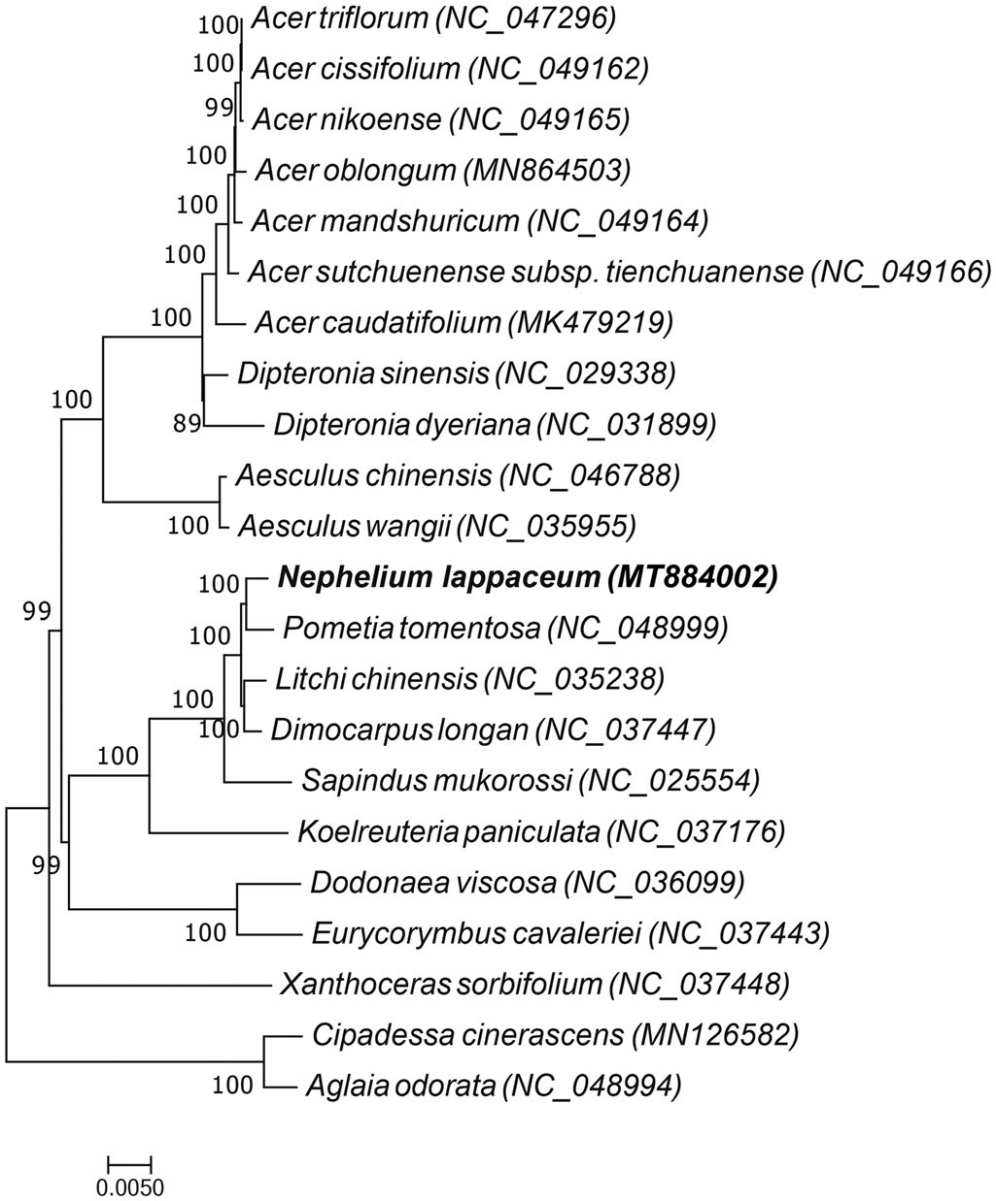
ML phylogenetic tree of *Nephelium lappaceum* and 19 other species of the Sapindaceae family, and *Aglaia odorata* and *Cipadessa cinerascens*, which belongs to the Meliaceae family, was used as the outgroup, the bootstrap value was set to 1000.

## Data Availability

The genome sequence data that support the findings of this study are openly available in GenBank of NCBI at (https://www.ncbi.nlm.nih.gov/) under the accession no. MT884002. The associated **BioProject**, **SRA**, and **Bio-Sample** numbers are PRJNA669909, SRR12904123, and SAMN16480967 respectively.
